# Liraglutide counteracts obesity and glucose intolerance in a mouse model of glucocorticoid-induced metabolic syndrome

**DOI:** 10.1186/1758-5996-6-3

**Published:** 2014-01-14

**Authors:** Liselotte Fransson, Cristiane dos Santos, Petra Wolbert, Åke Sjöholm, Alex Rafacho, Henrik Ortsäter

**Affiliations:** 1 Department of Clinical Science and Education, Södersjukhuset, Karolinska Institutet, Research Center Floor 3, 118 83, Stockholm, Sweden; 2 Department of Physiological Sciences, Center of Biological Sciences, Federal University of Santa Catarina, Florianópolis, Brazil; 3 Department of Internal Medicine, Södertälje Hospital, SE-152 86, Södertälje, Sweden; 4 Department of Biochemistry and Molecular Biology, College of Medicine, University of South Alabama, Mobile, AL, USA; 5 Research Unit, Södertälje Hospital, SE-152 86, Södertälje, Sweden

**Keywords:** Corticosterone, Diabetes, Liraglutide, Metabolic syndrome, Insulin, Beta-cell, Islet

## Abstract

**Background:**

Glucocorticoid excess is commonly associated with diabetogenic effects, including insulin resistance and glucose intolerance. The effects of the long-term glucagon-like peptide 1 receptor agonist treatment on the metabolic syndrome-like conditions are not yet fully elucidated. Thus, we aimed to test whether long-term liraglutide treatment could be effective as a therapy to counteract the metabolic dysfunctions induced by chronic glucocorticoid exposure.

**Methods:**

Mice were given corticosterone or vehicle via their drinking water for five consecutive weeks. In addition, mice were treated with once-daily injections of either PBS or liraglutide.

**Results:**

Liraglutide treatment slowed progression towards obesity and ectopic fat deposition in liver that otherwise occurred in corticosterone-treated mice. The drug reduced the increment in serum insulin caused by corticosterone, but did not affect the reduction of insulin sensitivity. Furthermore, liraglutide improved glucose control in mice exposed to corticosterone as evident by a delay in the progression towards post-prandial hyperglycemia and enhanced glucose clearance during a glucose tolerance test. Glucose-stimulated C-peptide levels were higher in those mice that had received liraglutide and corticosterone compared to mice that had been treated with corticosterone alone, indicating a positive role of liraglutide for beta-cell function. Morphometric analysis revealed increased beta- and alpha-cell masses that were associated with more Ki67-positive islet cells in corticosterone-treated mice irrespective of whether they were co-treated with liraglutide or not. Liraglutide had no discernible effect on alpha-cell mass.

**Conclusion:**

Liraglutide can be beneficial for subjects at risk of developing metabolic complications as a result of glucocorticoid excess.

## Background

During long term treatment with glucocorticoids (GCs), many patients develop various degrees of glucose intolerance, some progressing into frank diabetes, a condition commonly known clinically as “steroid diabetes” [1]. Additionally, worsening of glycemic control in patients with known type 2 diabetes mellitus (T2DM) on GC therapy is also a clinically well-known phenomenon. The reasons for the impaired glucose tolerance are probably multifactorial, and the precise nature of the mechanisms contributing remains elusive. The insulin-producing pancreatic beta-cell may be extra susceptible to GC excess, since both iatrogenic Cushing syndrome and GC-induced diabetes in animal models are associated with loss of glucose-stimulated insulin secretion (GSIS), and GC immunosuppressive treatment adversely affects islet transplantation outcome [2]. In animal models susceptible to GC treatment, dexamethasone exposure causes GLUT-2 degradation, thereby impeding beta-cell glucose sensing [3] and increases islet glucose cycling as a consequence of augmented glucose-6-phosphatase activity [4,5]. *In vitro*, GCs exert several negative effects on beta-cell function including reduced GSIS and increased alpha-2 adrenergic receptor response [6], increased activity of K_v_1.5 channel (repolarizing potassium channel) [7], endoplasmic reticulum dysfunction [8], and increased beta-cell apoptosis [9,10]. Since the human insulin gene contains GC-sensitive transcriptional elements [11], it may be susceptive to deleterious effects of GCs. Beta-cell susceptibility to GCs may also be relevant in the natural unfolding of diabetes, since mice overexpressing the GC receptor restricted to the beta-cell develop early beta-cell failure, glucose intolerance and later in life overt diabetes [4,5]. Humans with impaired beta-cell function (low insulin responders) are predisposed to become overtly diabetic during GC therapy [12]. Clinically, steroid diabetes or worsened glycemic control in diabetic subjects is usually treated with insulin injections, oftentimes mixtures containing a high proportion of a direct acting insulin analogue to curb prandial glycemia. However, such regime may result in undesirable side effects. The risk of incurring hypoglycemia, weight gain and adiposity -- on top of what is the result of GC therapy -- is a significant drawback of insulin treatment. Recently, another class of antidiabetic agents, incretin-based therapy, has been made available [13,14]. This novel treatment modality is based upon activation of the receptor for glucagon-like peptide 1 (GLP-1), which leads to enhanced GSIS, glucagon suppression and other antidiabetic effects [13,14]. GLP-1 is synthesized in enteroendocrine L-cells and is released post-prandially in proportion to caloric intake. Especially carbohydrate and fat seem to be effective stimuli for GLP-1 secretion [15]. GLP-1 drugs, in contrast to insulin, are devoid of risk for hypoglycemia and weight gain. Exendin-4, the first generation GLP-1 receptor (GLP-1R) agonist was recently shown to improve beta-cell function in healthy men treated for two days with prednisolone [16]. Similarly, Matsuo *et al*. reported on four cases of patients with type 2 diabetes with worsened glycemic control due to GCs who were successfully treated with exendin-4 administration [17]. Although these studies indicate beneficial glycemic effects of GLP-1 receptor activation after GC treatment, they neither address the ability of these drugs to counteract the long-term effects of GC treatment. In the present work, we aimed at addressing this issue in an animal model of steroid diabetes by using liraglutide, an efficacious second generation GLP-1 analogue in clinical use [18,19] and to study the mechanisms behind any protective effect exerted by liraglutide.

## Methods

### Animals and treatment

Experiments were performed on 8-week-old male C57Bl/6J mice (Nova, Sollentuna Sweden) which were fed *ad libitum* and housed in 12 hours of light/12 hours of dark cycles. The study was performed according to the guidelines of Karolinska Institutet and approved by the Stockholm South animal ethics committee (S49-12). Animals were randomly assigned to either receive corticosterone (100 μg/ml, [Sigma, St Louis, MO, USA]) or vehicle (1% ethanol) via their drinking water. This corticosterone regimen is known to produce a valuable mouse model with metabolic syndrome features [20,21]. In addition, half of the mice in each group were given once daily *s.c*. injections (between 9:00 and 10:00 am) of liraglutide (Novo Nordisk A/S, Bagsvaerd, Denmark) and the other half were given PBS in the same manner. To improve liraglutide tolerability, the drug was given in escalating doses starting at 0.15 mg per kg body weight with a daily increment of 0.025 mg/kg until the final dose of 0.3 mg per kg body weight was reached. This dose was maintained during the remaining part of the study period.

Food intake, body weight, and blood glucose levels in random-fed mice were monitored weekly, using a hand-held glucometer (One-Touch Ultra 2; LifeScan, Milpitas, CA, USA). Every week blood was collected for serum insulin level evaluations with ELISA (Mercodia, Uppsala, Sweden). Blood samples for determination of glucose and insulin concentrations were taken in the morning in non-fasted animals prior to administration of liraglutide or PBS. The total exposure period was five weeks during which the animals were subjected to insulin or glucose tolerance tests (IPinsTT and IPGTT, respectively) as described below. After the five weeks of treatment, mice were sacrificed and organs were collected. Pancreatic glands were dissected and fixated in 4% phosphate-buffered paraformaldehyde, paraffin-embedded and sectioned for immunohistochemistry. Fat deposits and spleen were gently removed and weighed, and liver fragments were snap-frozen for later sectioning and oil-red-O staining. Blood was collected by heart puncture for later analyses of serum non-esterified fatty acids (NEFA), total cholesterol and triglycerides (analyzed at Karolinska University Laboratory at Södersjukhuset, Stockholm and Center for Inherited Metabolic Diseases, Karolinska University Hospital, Solna) and for corticosterone (corticosterone EIA, Enzo Life Sciences, Lausen, Switzerland) and corticotropin (mouse corticotropin ELISA, Wuhan EIAab Science, Wuhan, China).

### IPinsTT and IPGTT

IPinsTT and IPGTT were performed during the fourth and fifth week of treatment, respectively. For IPinsTT and IPGTT, mice were fasted one or six hours, respectively, and then injected IP with insulin (2 IU/kg body weight) or glucose (1.5 g/kg body weight). On the day of the tolerance tests, liraglutide was injected as usual in the morning, 6 hours before the experiments. The fasting time before the IPinsTT was kept short to avoid hypoglycemia and counter regulatory hormone secretion. Mice received the standard injections of PBS or liraglutide on the same days as the tolerance tests were performed. Blood glucose was monitored before and after the insulin or glucose injections. During the IPGTT blood was also collected before and at 15 and 30 minutes post injection for determination of C-peptide levels, which were evaluated using ELISA (Alpco Diagnostics, Salem, NH, USA).

### Oil-red-O staining

Frozen fragments of liver were embedded in NEG-50 (Thermo Scientific, Waltham, MA, USA). Cryo-sections (12 μm) were obtained from different parts of the tissues and stained for neutral lipids using oil-red-O as previously described [22]. Quantification of lipid droplets was done in accordance with [22].

### Quantitative approaches in endocrine pancreas

To study the morphometric parameters of endocrine pancreas, 5-6 pancreatic glands from each group were excised and processed according to a previous description [23] except that 4% phosphate-buffered paraformaldehyde was used as fixative solution.

#### ***Immunostaining***

Islet distribution of insulin, glucagon and Ki67 were analyzed as previously described [23].

#### ***Alpha- and beta-cell mass***

Alpha-cell and beta-cell mass was determined by point counting morphometry on each pancreas section immunostained for either glucagon or insulin, as previously described [24]. Each section was systematically scored with a grid of 130 points (final magnification × 100) using the image processing and analysis software - ImageJ (freely available at http://rsbweb.nih.gov/ij/). The numbers of intercepts over beta-cells, endocrine non-beta-cells and exocrine pancreatic tissue were counted. The beta-cell relative volume was calculated by dividing the intercepts over beta-cells by the intercepts over the total pancreatic tissue; the beta-cell mass was then estimated by multiplying the beta-cell relative volume by the total pancreas weight. The same methodology was applied for counting alpha-cell mass in sections immunostained for glucagon.

#### ***Islet-cell proliferation***

Averaged islet-cell proliferation was obtained by counting total islet-cell stained for insulin and Ki67 using the same software cited above. All islets found in each pancreas section were sampled (1,714 ± 218 beta-cell nuclei per group). The rate of islet-cell proliferation was expressed as the proportion of Ki67-positive cells of total islet cells [24].

### Statistical analysis

Data are presented as mean ± s.e.m. The appropriate *t*-test, or one-way ANOVA followed by Bonferroni *post*-*hoc* test, was used as required to identify differences between groups, using GraphPad Prism 5.0 software. A value of p < 0.05 was considered statistically significant.

## Results

### Once daily injections of liraglutide delay body weight gain and obesity in corticosterone-treated mice

**Figure 1:**
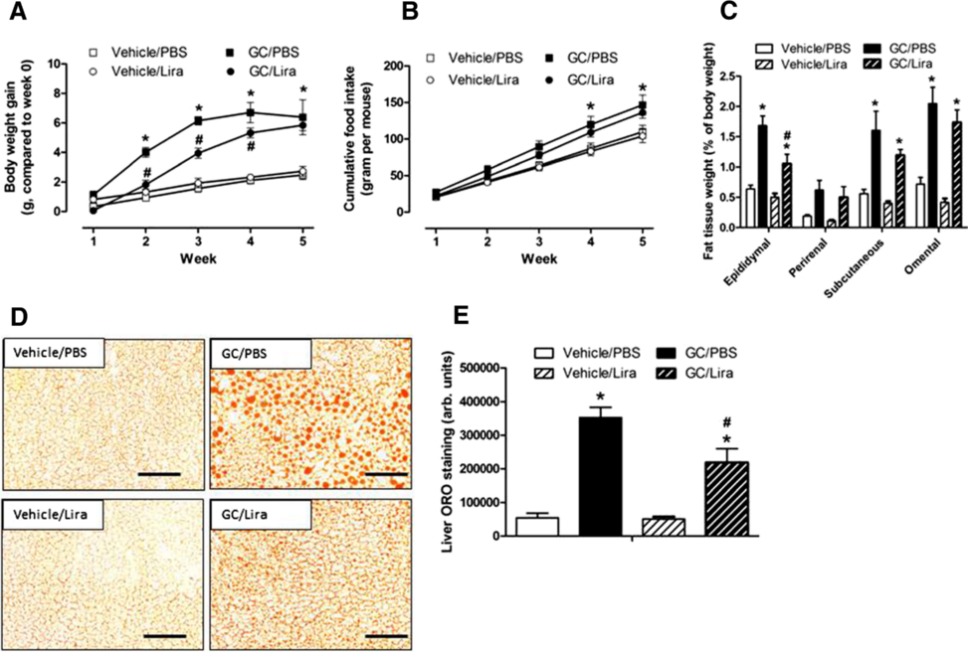
**Once daily injections of liraglutide delay body weight gain and obesity in corticosterone-exposed mice.** Male C57Bl/6J mice were treated with 1% ethanol (Vehicle, white squares and circles or white bars) or 100 μg/ml corticosterone (GC, black squares and circles or black bars) via the drinking water together with once-daily injections of either PBS (squares or unstriped bars) or liraglutide (Lira) at a final dose of 0.3 mg per kg body weight (circles or striped bars) for five consecutive weeks (n = 6). Body weight gain **(A)** and food intake **(B)** was determined weekly, fat deposit size **(C)**, and ectopic fat in liver **(D and E)** were determined after five weeks of treatment. Images in **(D)** are representative pictures of ORO staining in the examined tissue (20 × magnification, bar represents 150 μm). Data is shown as mean ± s.e.m. A * denotes a significant (p < 0.05) effect of corticosterone treatment and a # denotes a significant effect of liraglutide.

The present study includes four different experimental groups. Male C57Bl/6J mice were randomized to receive either vehicle (1% ethanol) or 100 μg/ml of corticosterone via their drinking water. In addition, both vehicle- and corticosterone-treated mice were given once daily injections of either PBS or liraglutide (final dose 0.3 mg/kg body weight). Vehicle-treated mice gained on average 2.5 ± 0.3 g weight over the five week study period. Mice receiving corticosterone via their drinking water had an augmented net body weight gain that was significant already after two weeks of treatment (Figure 1A) and was associated with a higher food intake (Figure 1B). Once daily injections with liraglutide did not influence food intake or body weight gain in vehicle-treated animals, but clearly slowed body weight gain in corticosterone-treated mice (Figure 1A). As expected, administration of corticosterone via the drinking water led to supraphysiological levels of the steroid hormone and suppressed endogenous levels of corticotropin (Table 1). Liraglutide treatment did not modulate serum levels of corticosterone or corticotropin neither in mice receiving vehicle nor corticosterone. This model of GC excess develops enlarged fat deposits and dyslipidemia (Figure 1C and Table 1). In concordance with a reduced body weight gain, fat deposit weights were generally lower in liraglutide-treated mice (Figure 1C). In contrast, treatment with liraglutide had minor, if any, effects on serum levels of NEFA, triglycerides and total cholesterol (Table 1). Chronic treatment with corticosterone is known to reduce spleen size [25]. In vehicle-treated mice that had received injections of PBS the spleen weight was 0.3 ± 0.04% of total body weight. Treatment with corticosterone reduced spleen weight to 0.13 ± 0.01% of total body weight (p < 0.01). However, once daily injections of liraglutide did not influence spleen weight in either vehicle- or corticosterone-treated mice, showing that liraglutide did not reduce corticosterone effects in mice in general. As corticosterone-exposed mice accumulate ectopic fat [26], which is known to contribute to deranged glucose control [27], we also monitored accumulation of neutral lipids in cryo-sections of liver tissue via oil-red-O staining. Indeed, corticosterone-treated mice had a 6.5-fold increase in liver oil-red-O staining compared to vehicle-treated mice (Figures 1D and E). While liraglutide had no effect on liver steatosis in vehicle-treated mice, the drug reduced hepatic neutral lipid content by approximately 40% in corticosterone-treated mice (Figures 1D and E). In conclusion, once-daily injections of liraglutide slow the progression towards obesity and ectopic fat deposition in liver that otherwise occurs during administration of corticosterone to mice.

**Table 1:** Serum parameters in mice

	**Experimental groups**			
	**Vehicle/PBS**	**GC/PBS**	**Vehicle/Lira**	**GC/Lira**
Corticosterone (ng/ml)	6.72 ± 2.29	46.56 ± 4.78*	7.83 ± 2.18	65.29 ± 10.6*
Corticotropin (pg/ml)	122 ± 14	45 ± 8*	134 ± 18	39 ± 14*
NEFA (mmol/L)	0.86 ± 0.04	1.30 ± 0.11*	0.92 ± 0.07	1.22 ± 0.14
Cholesterol (mmol/L)	2.84 ± 0.19	5.29 ± 0.46*	2.61 ± 0.12	4.16 ± 0.68*
Triglycerides (mmol/L)	1.26 ± 0.06	4.13 ± 1.10*	1.39 ± 0.15	2.98 ± 0.75

Male C57Bl/6J mice were treated with 1% ethanol (vehicle) or 100 μg/ml corticosterone (GC) via their drinking water together with once-daily injections of either PBS or liraglutide (Lira) at a final dose of 0.3 mg per kg body weight for five consecutive weeks (n = 5-6). At the end of the study, blood was collected by heart puncture and serum was obtained. Serum levels for the indicated parameters are shown as mean ± s.e.m. A * denotes a significant (p < 0.05) effect of corticosterone treatment.

### Liraglutide improves glucose tolerance in corticosterone-treated mice

**Figure 2:**
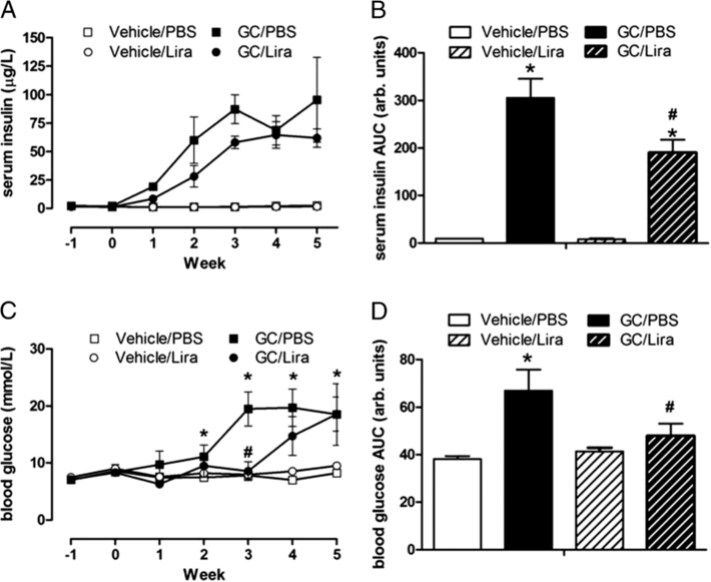
**Once daily injections of liraglutide delay hyperinsulinemia and hyperglycemia in corticosterone-exposed mice.** Male C57Bl/6J mice were treated with 1% ethanol (white squares and circles or white bars) or 100 μg/ml corticosterone (black squares and circles or black bars) via the drinking water together with once-daily injections of either PBS (squares or unstriped bars) or liraglutide at a final dose of 0.3 mg per kg body weight (circles or striped bars) for five consecutive weeks (n = 6). Serum insulin levels **(A and B)** and blood glucose levels **(C and D)** were determined every week in random-fed animals. Data is shown as mean ± s.e.m. A * denotes a significant (p < 0.05) effect of corticosterone treatment and a # denotes a significant effect of liraglutide.

**Figure 3:**
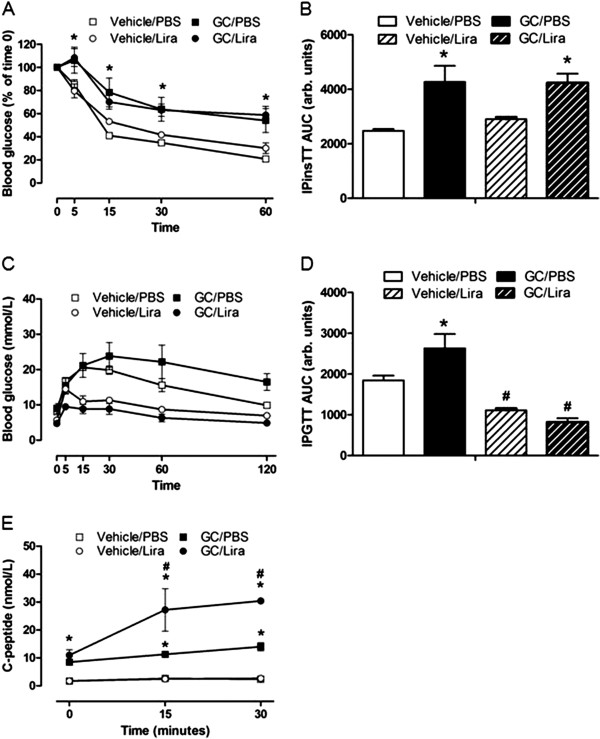
**Once daily injections of liraglutide do not affect insulin sensitivity but improve glucose tolerance in corticosterone-exposed mice.** Male C57Bl/6J mice were treated with 1% ethanol (white squares and circles or white bars) or 100 μg/ml corticosterone (black squares and circles or black bars) via the drinking water together with once-daily injections of either PBS (squares or unstriped bars) or liraglutide at a final dose of 0.3 mg per kg body weight (circles or striped bars) for five consecutive weeks (n = 6). After four weeks of treatment insulin sensitivity was determined via an IPinsTT **(A and B)** and after five weeks of treatment glucose tolerance was determined via an IPGTT **(C and D)**. During the IPGTT serum samples were collected and C-peptide levels were measured **(E)**. Data is shown as mean ± s.e.m. A * denotes a significant (p < 0.05) effect of corticosterone treatment and a # denotes a significant effect of liraglutide.

To investigate the effects of liraglutide on glucose control in corticosterone-treated mice, serum insulin and blood glucose levels were measured every week in random-fed mice throughout the study period. Corticosterone given via the drinking water time-dependently increased serum insulin levels (Figure 2A) as compared to vehicle-treated mice, indicating reduction of peripheral insulin sensitivity. Liraglutide attenuated circulating insulin levels by approximately 35% in corticosterone-treated mice (Figures 2A and B). Administration of corticosterone via the drinking water did not induce fasting hyperglycemia. The fasting blood glucose levels after five weeks of treatment were 8.1 ± 0.7 mmol/L and 9.0 ± 1.4 mmol/L in vehicle and corticosterone treated mice, respectively (not significant). In contrast, mice treated with corticosterone demonstrated impaired glucose control in the fed state. After two weeks of treatment with corticosterone, blood glucose levels in random-fed mice were significantly higher as compared to vehicle-treated mice and after three weeks of treatment the blood glucose levels were above 20 mmol/L in corticosterone-treated mice (Figure 2C). Once-daily injections of liraglutide improved glucose control in mice exposed to corticosterone. Fed blood glucose levels were normal in mice receiving liraglutide in combination with corticosterone up until the third week of treatment (Figure 2C). Integrated over the total five week study period the GLP-1R agonist reduced hyperglycemia in random fed mice by 30% (Figure 2D). However, after five weeks of GC exposure liraglutide was no longer able to prevent the hyperglycemia in this cohort of mice. To further evaluate the effects of liraglutide on glucose control in corticosterone-exposed mice we performed an IPinsTT and an IPGTT after four and five weeks of treatment, respectively. Treatment with corticosterone significantly reduced the blood glucose lowering effect of insulin (Figures 3A and B [B is AUC]). Treatment with liraglutide neither influenced insulin sensitivity in corticosterone- nor in vehicle-treated animals. Following IPGTT, glucose tolerance was reduced in corticosterone-treated mice as compared to vehicle-treated mice (Figures 3C and D [D is AUC]). Once daily injections of liraglutide significantly improved glucose tolerance in both vehicle- and corticosterone-treated animals, with the largest effect obtained in corticosterone-treated mice (Figures 3C and D). C-peptide levels during the IPGTT were analyzed, showing that before glucose injection both cohorts of corticosterone-treated mice had higher levels of C-peptide as compared to their respective control groups, which reflects the higher basal insulin levels (Figure 3E). During the *in vivo* GSIS (IPGTT), the C-peptide levels were modestly increased in mice receiving corticosterone, 33 ± 6% above basal at 15 minutes and 65 ± 17% above basal at 30 minutes post glucose injection (Figure 3E). In contrast, the glucose-stimulated increase in serum C-peptide levels in mice treated with corticosterone and liraglutide was 149 ± 30% and 178 ± 23% above basal at 15 minutes and 30 minutes post glucose injection, respectively, indicating an improvement of beta-cell responsiveness to glucose by liraglutide treatment (Figure 3E).

### Liraglutide has no effect on the increased beta-cell mass induced by corticosterone

**Figure 4:**
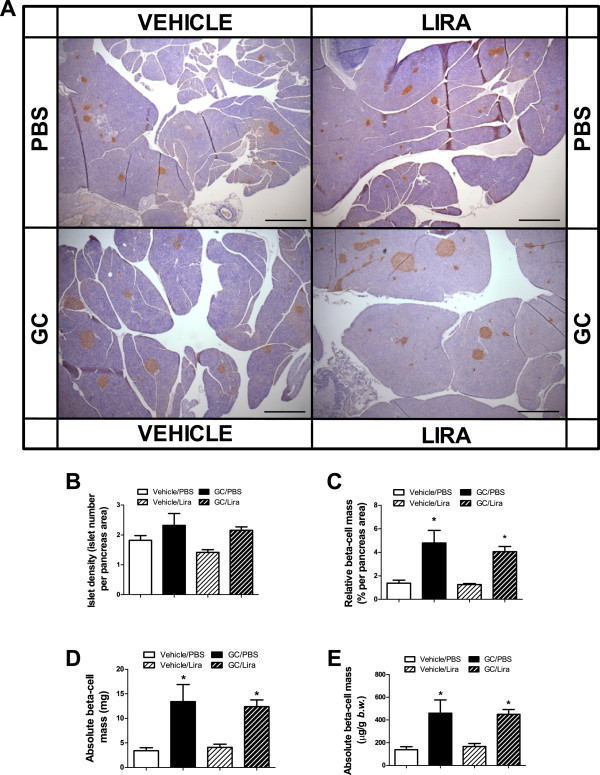
**Once daily injections of liraglutide do not affect the increment of pancreatic beta-cells mass seen in corticosterone-exposed mice.** Male C57Bl/6J mice were treated with 1% ethanol (vehicle, white bars) or 100 μg/ml corticosterone (GC, black bars) via the drinking water together with once-daily injections of either PBS (unstriped bars) or liraglutide (Lira) at a final dose of 0.3 mg per kg body weight (striped bars) for five consecutive weeks (n = 5-6). After treatment the pancreatic glands were dissected and processed for histology. Tissue sections were immunostained for insulin-positive cells **(A)**. Morphometric analysis was used to determine islet density **(B)**, relative beta-cell mass **(C)** and absolute beta-cell mass either expressed in mg **(D)** or as μg per g body weight **(E)**. Images in **(A)** are representative pictures of insulin staining in the examined tissue (40 × magnification, bar represents 500 μm). Data is shown as mean ± s.e.m. A * denotes a significant (p < 0.05) effect of corticosterone treatment.

**Figure 5:**
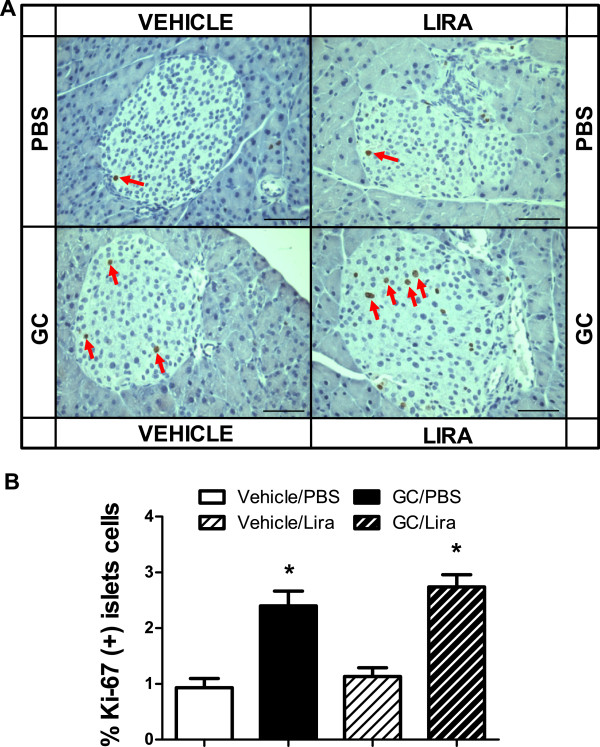
**Once daily injections of liraglutide do not affect beta-cell proliferation in corticosterone-exposed mice.** Male C57Bl/6J mice were treated with 1% ethanol (vehicle, white bars) or 100 μg/ml corticosterone (GC, black bars) via the drinking water together with once-daily injections of either PBS (unstriped bars) or liraglutide (Lira) at a final dose of 0.3 mg per kg body weight (striped bars) for five consecutive weeks (n = 5-6). After treatment the pancreatic glands were dissected and processed for histology. Tissue sections were immunostained for Ki67-positive cells **(A, red arrow)**. Morphometric analysis was used to the percentage of beta-cells that stained positive for Ki67 in the different mice cohorts **(B)**. Images in **(A)** are representative pictures of Ki67 staining in the examined tissue (400 × magnification, bar represents 50 μm). Data is shown as mean ± s.e.m. A * denotes a significant (p < 0.05) effect of corticosterone treatment.

**Figure 6:**
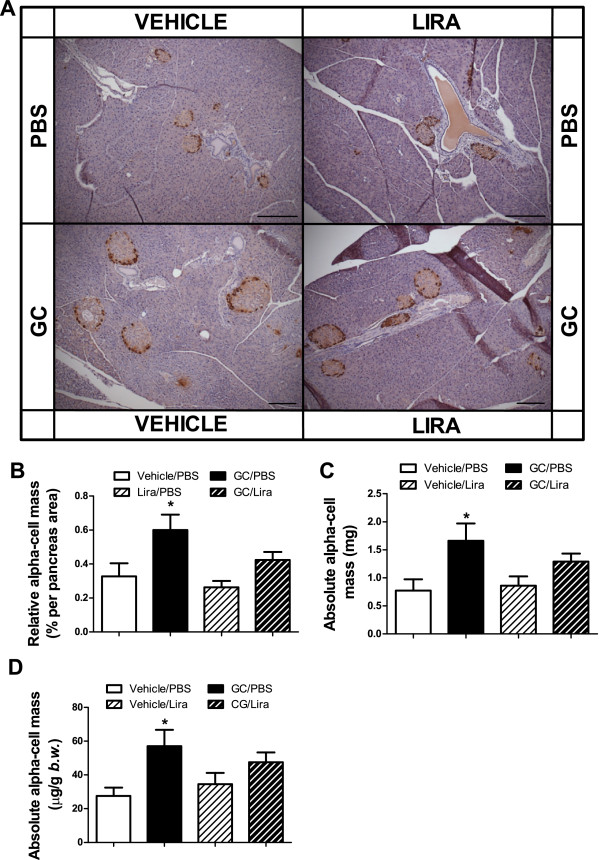
**Corticosterone-exposed mice display increased alpha-cell mass.** Male C57Bl/6J mice were treated with 1% ethanol (white bars) or 100 μg/ml corticosterone (GC, black bars) via the drinking water together with once-daily injections of either PBS (unstriped bars) or liraglutide (Lira) at a final dose of 0.3 mg per kg body weight (striped bars) for five consecutive weeks (n = 5-6). After treatment, the pancreatic gland was dissected and processed for histology. Tissue sections were immunostained for glucagon-positive cells **(A)**. Morphometric analysis was used to determine relative alpha-cell mass **(B)** and absolute alpha-cell mass either expressed in mg **(C)** or as μg per g body weight **(D)**. Images in **(A)** are representative pictures of glucagon staining in the examined tissue (100 × magnification, bar represents 200 μm). Data is shown as mean ± s.e.m. A * denotes a significant (p < 0.05) effect of corticosterone treatment.

To investigate possible structural changes in the endocrine pancreas in the present insulin-resistant mice model, we determined the beta- and alpha-cell masses in histological sections of pancreatic glands taken after five weeks of treatment with or without corticosterone in the absence or presence of liraglutide. The total pancreas weight was not altered among the groups (data not shown) nor was there any significant difference between the groups in the number of islets per pancreatic gland (Figures 4A and B). Instead there was an apparent islet hypertrophy in both groups of corticosterone-treated mice (Figure 4A). Morphometric analysis revealed that the relative and absolute beta-cell mass was significantly increased in pancreata obtained from corticosterone-treated mice irrespective of whether they were co-treated with liraglutide or not (Figures 4C-E). This increase in beta-cell mass is explained by enhanced islet-cell proliferation (most likely beta-cell proliferation) in corticosterone-treated mice. Figure 5A shows a higher distribution of Ki67-positive nuclei in both corticosterone-treated groups compared with their respective control groups. The islet-cell proliferation expanded significantly, reaching 2.6- and 2.4-fold increase in mice receiving corticosterone in the absence or presence of liraglutide treatment, respectively, compared to their respective controls (Figure 5B). Our analysis of alpha-cell mass in histological sections of pancreata taken after five weeks of treatment, with or without corticosterone in the absence or presence of liraglutide, showed a significant increase in alpha-cell mass in corticosterone-treated mice (Figure 6). Liraglutide *per se* had no significant effect on any of the alpha-cell parameters evaluated, but seemed to cause a partial attenuation in the corticosterone-induced expansion of alpha-cell mass. Altogether, these data demonstrate that corticosterone administration to mice induces expansion of beta-cell and alpha-cell masses. Liraglutide exerted no positive or negative effect upon beta-cell mass *per se* or when combined with corticosterone. For alpha-cell mass, however, liraglutide treatment seems to result in a partial attenuation of the absolute alpha-cell mass expansion caused by corticosterone treatment.

## Discussion

The main finding in this paper is that once daily injections with the DPP-4-resistant GLP-1R agonist liraglutide delay the development of hyperglycemia and promote beta-cell function in a mouse model of GC-induced metabolic syndrome. In this model, corticosterone is administrated via the drinking water. The model recapitulates in many respects the metabolic syndrome in humans, including body weight gain, dyslipidemia, ectopic lipid deposition and hypertension that occurs in conjunction with insulin resistance and glucose intolerance [20,21]. It should, however, be noted that – after five weeks of treatment with corticosterone – the mice became catabolic as evident by reduced body weight and that liraglutide was not able to fully protect against the diabetogenic effects of the GC in this setting. Thus, liraglutide fell short in providing full protection against corticosterone-induced glucose tolerance which of course limits the value of this study.

Nevertheless, it is clear that liraglutide postponed the appearance of obesity, hyperinsulinemia and hyperglycemia. It is also clear that administration of the GLP-1R agonist improved glucose clearance in an IPGTT. We could exclude the possibility that liraglutide caused any general reduction in peripheral GC sensitivity, as there were neither any differences between the effect of corticosterone on the glucose decay during the insulin tolerance test nor any impact of liraglutide on the reduced spleen size incurred by GC treatment. As nausea and vomiting are reported side effects during liraglutide treatment [28], this would be a potentially confounding factor in this study. However, we took care to avoid these side effects by slowly increasing the dosage until the final dose of liraglutide was reached and as indicated by the similar food intake in liraglutide-exposed mice as compared to mice treated with corticosterone or vehicle this dosing regime was successful. Liraglutide has previously been shown to efficaciously curb glucose intolerance and diabetes in both animal models [29] and in human T2DM patients [30]. However, its potential to counteract the diabetogenic effects of long-term steroid treatment has not previously been rigorously tested. Our findings show that liraglutide can delay hyperglycemia and avoid glucose intolerance that otherwise occurred as a consequence of steroid treatment. Thus, liraglutide may confer treatment benefits in patients at increased risk of developing steroid-induced diabetes. Liraglutide treatment was initiated simultaneously to corticosterone and our data supports that such an approach should be considered when handling patients with susceptibility towards steroid-induced diabetes, like subjects with *a priori* known insulin resistance [31], low insulin secretion capacity [12,32], genetic predisposition [33], or in patients with obesity [34]. Our findings support a recent case report where exenatide (another GLP-1R agonist) improved glucose control in patients with T2DM with worsened glycemic control caused by GC therapy [17], and yet another randomized, placebo-controlled, double-blind, crossover exploratory study in eight healthy men where it was shown that exenatide prevented GC-induced glucose intolerance and islet dysfunction [16]. However, we think that the experimental data presented here advances the field in several aspects. First, the GC exposure time used in this study is extended to last for several weeks and in this aspect it better mimics the treatment time for patients prescribed GC-based drugs. Second, we show that once-daily injections with the DPP-4-reistant GLP-1R agonist liraglutide can improve glycemic control in this model. Finally, as will be discussed below, we discriminate between two aspects of beta-cell adaptation to GC induced insulin resistance. Liraglutide treatment may also reduce stress responses in rodents [35], an effect that can contribute to the anti-diabetic observed in our study.

This study also reports on the ability of liraglutide to counteract GC-induced obesity. As expected, abdominal obesity developed in corticosterone-treated mice. Importantly, liraglutide delayed weight gain in corticosterone-treated mice, which was associated with reduced epididymal fat mass. A role of GLP-1 in obesity pathophysiology was suggested already in 1983 when it was shown that L-cell secretory activity was reduced in morbidly obese subjects [36]. Furthermore, obese patients with reduced post-prandial GLP-1 secretion displayed improved GLP-1 secretory activity upon weight loss [37]. This may be a consequence of lipotoxic actions directed towards the L-cells as these cells succumb by lipoapoptosis when exposed to palmitate in culture [38]. As GC therapy specifically induced visceral obesity, which is much more prone to induce insulin resistance than subcutaneous adiposity, it is additionally important that, as showed herein, liraglutide delays the progression towards obesity that otherwise relentlessly occurs during long-term GC treatment. These findings are in concordance with those reported in the LEAD (Liraglutide Effect and Action in Diabetes) studies series, where the weight loss after 30 week to 52 week treatment with liraglutide was 2–3 kg more than placebo treatment [39-44]. The weight loss occurred in the ratio 2:1 between fat and lean tissue, and the relative weight loss was greater in visceral fat compared with subcutaneous adipose tissue [45]. Also hepatic steatosis was reduced in patients during treatment with liraglutide [45], which supports our findings with decreased liver neutral lipid content in corticosterone-treated mice receiving liraglutide.

The present study also investigates the plasticity of the pancreatic beta-cell and alpha-cell mass in this setting of GC excess. There is a strong correlation between insulin sensitivity and insulin secretion such that reduced insulin sensitivity leads to augmented insulin secretion in order to maintain glucose control [21]. Compensatory hyperinsulinemia in response to GC-induced reduction of insulin sensitivity may be attributable to increased beta-cell responsiveness to glucose and/or increased beta-cell mass [24,28]. In response to corticosterone treatment the beta-cell mass increased as a consequence, at least in part, of enhanced beta-cell proliferation. This observation is in agreement with the well-established reciprocal relationship between insulin sensitivity and pancreatic islet function. As insulin sensitivity decreases, the insulin secretion initially and adaptively increases in order to maintain the control of glucose homeostasis [46]. The compensatory increase of circulating insulin in response to the GC-imposed insulin resistance resulted from some beta-cell adaptation that includes augmented beta-cell function [47] and/or beta-cell mass [48]. Mice treated with corticosterone and liraglutide had the same impairment of insulin sensitivity as indicated by their similar capacity to clear glucose after an injection with insulin (Figures 3A and B). Mice treated with corticosterone with or without liraglutide also displayed the same increase in beta-cell mass (Figure 4), indicating that the beta-cell mass is primarily a reflection of the prevailing reduced insulin sensitivity and that liraglutide is not predominantly targeting insulin sensitivity.

An increased beta-cell mass in corticosterone-treated mice seems to be sufficient to maintain fasting normoglycemia, since this parameter was not affected after five weeks of treatment with the steroid. In the fed state, however, the insulin secretion in response to food intake was not enough to avoid the appearance of hyperglycemia, despite the higher beta-cell mass observed in corticosterone-treated groups. Liraglutide had no effect on beta-cell mass but displayed a marked ability to improve glucose clearance in response to a glucose challenge (IPGTT, Figure 3). These observations point towards an acute effect of liraglutide so that the optimal response to glucose challenge in both groups of mice treated with liraglutide is explained by short-term liraglutide effects. Our finding of augmented glucose-stimulated C-peptide secretion in liraglutide-treated mice indicates a direct acute effect on pancreatic beta-cells that may turn beta-cells more competent to sense glucose. A puzzling observation is that liraglutide improved glucose tolerance also in vehicle-treated mice despite similar insulin sensitivity and glucose-stimulated C-peptide secretion. Since C-peptide clearance occurs mainly through renal mechanisms, and GLP-1 is known to positively impact renal function [49] we cannot exclude that increased insulin secretion may have been masked by increased renal clearance of C-peptide. Thus, the positive effects of liraglutide on glucose tolerance in this model of metabolic syndrome are best described by a direct effect on pancreatic beta-cells that seems to promote augmented exocytosis rather than promoting beta-cell proliferation and/or acting on peripheral insulin action. This means that liraglutide would be most effective if given at an early stage of T2DM when beta-cell mass is preserved. A recent publication reported on a positive association between incretin-based therapy and an expansion of pancreatic alpha-cell mass [50] in human *post mortem* specimens. However, we did not observe any increase in alpha-cell following 5 weeks of liraglutide treatment. In fact, liraglutide reduced the induction of alpha-cell mass seen in corticosterone-treated mice, although this finding did not reach statistical significance.

## Conclusions

In this paper we show that the GLP-1R agonist liraglutide delays progression towards obesity and prandial hyperglycemia and efficiently enhances *in vivo* GSIS in a mouse model of GC-induced metabolic syndrome. Thus, GLP-1R agonists may offer advantages for patients suffering from steroid diabetes or glucose intolerance as a side effect of long-term GC therapy.

## Competing interest

Å.S. has received research grants, consultancy fees, lecture honoraria, and fees for expert testimony from Novo Nordisk, the manufacturer of liraglutide. The other authors declare that they do not have any competing or financial interests.

## Authors’ contribution

LF, ÅS, AR and HO conceived the hypothesis and designed the experiments. LF, CS, PW and HO performed the experiments. LF, CS, PW, AR and HO analyzed the data. LF, ÅS, AR and HO wrote and edited the paper. All authors have read and approved the final version.
